# Measuring Outcomes in Adult Weight Loss Studies That Include Diet and Physical Activity: A Systematic Review

**DOI:** 10.1155/2014/421423

**Published:** 2014-11-25

**Authors:** Rachel A. Millstein

**Affiliations:** SDSU/UCSD Joint Doctoral Program in Clinical Psychology, 6363 Alvarado Court, San Diego, CA 92120, USA

## Abstract

*Background*. Measuring success of obesity interventions is critical. Several methods measure weight loss outcomes but there is no consensus on best practices. This systematic review evaluates relevant outcomes (weight loss, BMI, % body fat, and fat mass) to determine which might be the best indicator(s) of success. *Methods*. Eligible articles described adult weight loss interventions that included diet and physical activity and a measure of weight or BMI change and body composition change. *Results*. 28 full-text articles met inclusion criteria. Subjects, settings, intervention lengths, and intensities varied. All studies measured body weight (−2.9 to −17.3 kg), 9 studies measured BMI (−1.1 to −5.1 kg/m^2^), 20 studies measured % body fat (−0.7 to −10.2%), and 22 studies measured fat mass (−0.9 to −14.9 kg). All studies found agreement between weight or BMI and body fat mass or body fat % decreases, though there were discrepancies in degree of significance between measures. *Conclusions*. Nearly all weight or BMI and body composition measures agreed. Since body fat is the most metabolically harmful tissue type, it may be a more meaningful measure of health change. Future studies should consider primarily measuring % body fat, rather than or in addition to weight or BMI.

## 1. Introduction

The obesity treatment literature includes many sophisticated analyses, methods, and conclusions, yet the problem persists [1–3]. Given all of the information now known, to move forward in intervention development and evaluation, more accurate measures of success are needed to monitor changes. The field of obesity treatment often has redundancy of interventions and measures but heterogeneity of outcome measures, making it difficult to combine results and move toward the ultimate goal of achieving healthy weights [3–5]. Most weight loss studies measure weight and/or BMI to assess intervention-related changes, given their ease of measurement and interpretation [4, 5]. Though well correlated with body composition, weight and BMI only inform total loss or change, which could include lean body mass in addition to fat loss [6, 7]. BMI may also inaccurately reflect intervention-related change, in that it does not account for bone and muscle density, frame size, and fat distribution [8, 9].

Loss of fat is the major outcome of interest for a variety of health reasons. Body fat can have varying degrees of benefit and harm, depending on location, amount, and time of fat disposition [10–12]. While babies and young children depend on body fat to promote brain and tissue growth, as children age, the percent of fat that is beneficial decreases [13]. Among older adults, body fat is also metabolically harmful, with an exception that it may offer some protection against bone loss and fractures [14]. However, for adults, excess body fat is widely acknowledged to be associated with such diseases as type 2 diabetes, stroke, hypertension, cardiovascular disease, and arthritis [15, 16]. In particular, abdominal fat has been associated with increased morbidity and mortality among adults [11, 12]. Body fat is best captured by measures other than weight and BMI, such as bioelectrical impedance (BIA), dual-energy X-ray absorptiometry (DEXA), underwater weighing, air displacement, and skinfold thickness [17]. Measuring body fat as part of weight loss interventions is a common but not universal practice and it is usually viewed as a secondary outcome, with weight or BMI being primary [5, 18].

Compounding the measurement issues described above, adult weight loss intervention studies primarily target dietary interventions, rather than (or more than) physical activity (PA) interventions [5]. Although diet can lead to reduced body fat, it can also lead to overall weight loss that can include reduced fat-free mass (bone and muscle) [19]. Overall weight loss does not distinguish between types of tissue mass lost. Much research shows that PA is a key driver of fat loss and maintenance or increase of fat-free mass [19, 20]. Ideally, interventions should include components of both diet and PA, to reduce body fat and maintain or increase fat-free mass [18, 20]. Therefore, studies that include PA as a component of interventions (in addition to diet) should theoretically include a measure other than weight or BMI to potentially best capture changes.

The objectives of this review were to address the following questions:

(1) What are the best or most consistent measures of success in adult weight loss interventions that include diet and PA: weight or BMI, or body composition? (2) Are weight loss or BMI changes or body composition changes adequate measures of intervention success? (3) Do the studies that include a body composition measure in addition to weight or BMI reach the same conclusions? A systematic review was conducted to examine and evaluate all of the existing findings on these topics. This review does not include a meta-analysis because intervention studies contain many heterogeneous components (duration, intensity, length, and setting of interventions), so that compiling results may lead to incomplete or inconclusive findings.

## 2. Methods

### 2.1. Search Strategy

The Cochrane library was searched for existing reviews on this topic and information from related systematic reviews. As there were no available or registered review papers on these specific questions, the following search methods were employed in July and August 2012. This search was primarily conducted using online scientific literature databases. PubMed (1953–present) and PsychInfo (1806–present) were used to do an exhaustive search combining all terms. Additional search resources were used to acquire remaining papers meeting review criteria: the online database at Google Scholar (1992–present) and searching through references from eligible papers found (ancestry search). The overarching goal of the search was to identify studies of adult weight loss interventions that included diet and PA and a measure of weight or BMI and body composition. The following title and abstract keyword search terms were used in all databases, with limitations to humans, English language, randomized controlled trials (RCTs) or clinical trials, and adults over age 18: “weight loss” AND “overweight” OR “obese” OR “obesity” AND “diet” OR “dietary” OR “calorie restriction” AND “exercise” OR “physical activity” OR “fitness” AND “BMI” OR “body mass index” AND “fat loss” OR “body composition” OR “skinfold” OR “skinfold thickness” or “tricep skinfold” OR “DEXA” OR “DXA” OR “underwater weigh^*^” OR “bioelectrical impedance” OR “BIA.”

### 2.2. Study Selection

Eligibility criteria for inclusion into this review were as follows. Study types were limited to randomized controlled trials published in English. There was no lower limit on year of publication. The types of participants intended for this review were adults ages 18–65 who were overweight or obese (BMI ≥ 25.0). Studies of adults with comorbid health conditions (i.e., prediabetes and prehypertension) were included because many interventions target such populations. All interventions were eligible if they targeted weight loss and included a diet and aerobic PA component. No restrictions were placed on duration, intensity, or setting of intervention (i.e., inpatient and outpatient). The eligible outcome measures were at least one measure of overall weight change (taken both before and after intervention), pounds or kilograms of weight or BMI reduction, and at least one body fat measure (taken both before and after intervention): skinfold thickness, DEXA, underwater weighing, BIA, or air displacement. While waist or hip circumference is sometimes considered to be a body composition measure, it can be too gross to reliably identify changes in short-term studies, so it was excluded from this search.

Exclusion criteria were studies not published in English, inclusion of children or older adults, interventions that included only diet or only PA, interventions in which the only PA was strength training, studies that included only one outcome measure (weight or BMI or body composition), nonintervention studies or designs other than RCTs (reviews, position papers, and cross-sectional or noncontrolled studies), studies that included dietary supplements or drugs to assist in weight loss, secondary or redundant data analyses (in that case, only the primary results were included in this review), and the case when the full-text article was unavailable from interlibrary loan, online sources, or correspondence with the author.

The study selection process, all completed by the author, began with general keyword (title and abstract) searches in the databases and reference lists of appropriate papers. Of those titles that appeared relevant, a more thorough abstract review was conducted. Of abstracts that appeared relevant, a full-text review was conducted, when available, and all eligible full-text articles were included in this paper. Throughout the study selection process, duplicates were removed. If one trial published multiple papers, only the primary outcome paper or the most recent (whichever was most relevant) was included.

### 2.3. Data Extraction and Assessment of Studies

The author extracted and compiled the detailed data items and study characteristics from all articles. The data extraction tables included, as available, study type, sample size including gender breakdown, baseline ages, SES or race, comorbidities, intervention setting and length, body composition measure, baseline weight and/or BMI (and standard deviations (SD) or range), baseline body composition (and SD or range), follow-up weight and/or BMI (and SD), and follow-up body composition (and SD or range). For the principle summary measures, the variables were extracted from the studies when the data were published, but when the average weight and/or BMI change, average body composition change, and summary of agreement were not explicitly listed, they were calculated from the available data (e.g., calculating the average weight change from the baseline to follow-up weight).

## 3. Results

### 3.1. Study Selection

**Figure 1:**
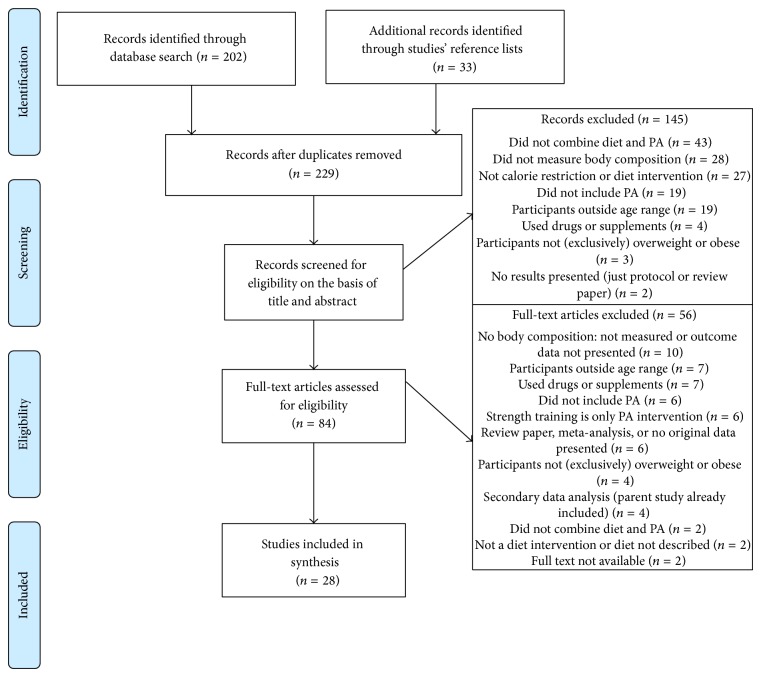
PRISMA flow diagram of article screening and eligibility.

The flowchart describing study screening, exclusion, eligibility, and selection is shown in Figure 1. Two hundred and thirty-five studies were identified through the various search sources. Of the nonduplicate studies, 145 were excluded for the reasons listed in the flowchart; the main reasons were that the intervention did not include both diet and PA, that the study did not include or measure body composition, and that the intervention did not involve a calorie restricted diet (e.g., manipulating macronutrient composition in isocaloric weight maintenance diets). Of the 56 excluded full-text articles, the main exclusion reasons were that the studies did not report body composition data, included older adults, and used drugs or supplements to aid weight loss. Ultimately, 28 studies met all inclusion criteria and were included in this review.

### 3.2. Study Characteristics

The study characteristics are presented in Table 1. There was much heterogeneity across studies, with respect to other characteristics. Sample sizes ranged from 5 to 111, and a majority of studies (18 studies = 64%) used only women participants. The studies included and focused on a range of adult ages, with baseline ages ranging from 28 to 54.7. No studies reported participants' socioeconomic status (SES). Of the few studies that reported racial composition of samples, most participants were white. The country of origin could perhaps be used to infer racial composition of samples when otherwise not indicated (e.g., primarily white participants in Belgium, The Netherlands, and Australia). Most studies were conducted in the United States, with fewer from Europe and Australia. Three main body composition measures were used (categories were not mutually exclusive, as some studies used multiple measures): DEXA was used in 13 studies, BIA was used in eight, and underwater weighing was used in six. Two studies used skinfold thickness (bi- or triceps), one used air displacement, and one used doubly labeled water with appropriate body composition calculations. Fifteen studies reported attrition rates, with values ranging from 2.5 to 48.8% loss.

The studies also demonstrated heterogeneity in factors such as intervention emphases, diet types, and length (Table 1). Most studies took place at outpatient facilities (university or hospital clinical research centers), two studies took place in inpatient clinics, and three studies did not give details on intervention setting. The interventions ranged from six weeks to two years in length, with half (13 studies) in the 12- to 16-week range, and five studies lasted 24 weeks. There were varied emphases and strategies for the different components of the interventions. For the diet component of the interventions, strategies included visits with a dietician, calorie restriction (ranges: 420–1800 cal/day), specific macronutrient proportions (e.g., % calories from fat and carbohydrates), liquid diets with and without solid food supplementation, food provided by programs, and nutrition education. For the calorie restriction, the most common target value was 1200 calories/day or a range including 1200, but the liquid and inpatient diets were much lower (e.g., 420 calories/day), and when men were included in studies, the calorie ranges were higher (1500–1800 calories/day). Several studies did not report a specific calorie target but rather gave each participant an individualized goal based on their resting metabolic rate and subtracting 250–1000 calories from that as the daily goal. For the PA components of the interventions, the strategies included varied amounts of activity per day and week, structured and supervised aerobic activity (commonly walking and indoor cycling), circuit classes, skills-based and noncompetitive activity classes, individualized heart rate training goals, and emphases on lifestyle activity. Most of the studies included weekly or biweekly individual or group meetings with a nutritionist, exercise counselor, and/or psychologist for support, education, and behavior modification strategies.

### 3.3. Outcome Measures

The outcome measures of baseline and follow-up BMI, body fat, and fat mass, plus the average changes, summary of agreement, and attrition are presented in Table 2. The follow-up outcomes are reported for the intervention group and for the longest follow-up period reported in the paper.

*Weight and BMI Outcomes.* Averages are presented as overall values, unadjusted for length or intensity of intervention and unweighted for sample size. All 28 studies reported weight as an outcome, and all reported losses with wide ranging values (−2.9 to −17.3 kg). The average weight loss at the longest reported follow-up time point was −8.2 kg. While most studies measured baseline BMI, only nine reported follow-up BMI values as an outcome. The range of BMI losses was −1.1 to −5.1 kg/m^2^, with an average loss of −3.1 kg/m^2^.

*Body Composition Outcomes*. Twenty studies measured % body fat, all finding losses (−0.7 to −10.2%), with an average decrease of −5.1%. Twenty-two of the studies measured fat mass, all finding losses. The range of fat mass lost was −0.9 to −14.9 kg, and the average decrease was −6.6 kg.

### 3.4. Agreement between Weight and Body Composition Measures

All of the studies found a decrease in weight, and those that measured BMI showed decreases that were in agreement with the weight losses. That is, there was no discrepancy between the interpretations of weight and BMI changes, though weight loss showed greater changes than BMI. All of the studies found a decrease in the body composition measure(s) used. Most of the weight lost was accounted for by fat loss. In control groups that included diet only interventions, the percentage of fat lost was significantly greater in the diet + PA groups, while the diet + PA groups preserved or increased their fat-free mass more so than control (diet only) groups. Overall, all of the studies had agreement between the weight or BMI and body composition measure(s). Two studies were discrepant between the significance of the measures: one had a borderline significant reduction in fat mass, with a significant weight decrease, and the other had a more significant reduction in fat mass and % body fat than weight. The details and significance of the agreement across outcomes are presented in Table 2.

Overall agreement did not describe the variability among weight loss measures completely. Across studies, the losses of fat mass and % body fat were proportionally greater than losses of BMI or weight and, in one instance, fat mass or % body fat losses were more significantly different before and after intervention than the overall weight or BMI losses. While there is some redundancy across weight loss measures, body composition was a consistent measure of success in these studies, with all of the studies that reported it finding decreases after intervention in parallel or more so than weight or BMI decreases.

## 4. Discussion

This review focused on evaluating measures of success in diet and PA weight loss interventions for adults. There was heterogeneity across studies in terms of designs, settings, participants, and types of outcome measures. Despite the differences, several themes emerged. All of the studies demonstrated agreement across measures (weight or BMI and % body fat or fat mass). However, nuances were present as well. Specifically and as expected, fat accounted for most of weight lost. While not a primary focus of this review, several studies reported that fat-free mass was preserved or increased following the interventions that included PA. The conclusions from measures of weight or BMI and body fat % or fat mass were largely in agreement and body fat is more metabolically informative than overall weight. Thus, it can be proposed that measuring body fat should be considered a primary outcome of weight loss studies. In aggregate, the data presented here support the conclusion that measuring % body fat or fat mass before and after weight loss interventions that include diet and PA may be the most efficient and informative measure of success or change.

Body fat measures are highly accurate, though each type has pros and cons. Underwater weighing is the gold-standard, but it can be unpleasant and inconvenient and often not feasible for obese participants [17]. It is rarely used in more recent studies, given the technological advances. DEXA, which was commonly used in the studies reviewed here, is highly accurate but expensive [17, 21, 22]. Cost is the most common barrier against using DEXA. However, given the increased precision of measurement, the cost of DEXA scans may be justifiable for research groups, with long-term use. BIA is also commonly used to measure fat, but its accuracy is more questionable and likely depends on different equations used to estimate body composition and the quality of the equipment [17, 23]. Skinfold thickness is an inexpensive and useful method for large surveillance studies, but its accuracy and reliability are variable, depending on rater training and precision of caliper location [17, 23]. Body fat also is the most metabolically harmful tissue type, so it makes sense to promote its measurement over others [11, 12, 15, 16]. Further, just as weight and BMI do not provide nuanced measures of health, body fat mass is a similarly gross measure. Body fat % may be a more indicative measure of health, as it allows more specificity by accounting for other tissue types' contributions to weight and body composition.

As BMI and weight are ubiquitous in weight loss studies [5], it is not likely that a paradigm shift will occur quickly, in which measurement shifts to focus on % body fat. Further, most people do not know their body fat percentage or have a context for its interpretation the way people do for weight and, increasingly, BMI. However, healthy body fat % ranges do exist for different ages and genders [13, 24], and these values could become more commonly evaluated and discussed. The evidence presented herein suggests that % body fat should become more of a primary measure of health and weight loss success, as it provides a succinct and meaningful indication of a person's body composition, and likely disease risk, than weight, BMI, and fat mass.

This review had several limitations. First, only studies available in English were included. While this may introduce bias, most of the countries that bear the largest burden of adult obesity are economically developed and English-speaking. So it is unlikely that many contradictory or critical studies have been published in other languages. As with all reviews, this one encompassed studies with considerable heterogeneity of study/intervention and outcome components. While, at this stage of research, this problem is mostly inevitable, future studies may become more homogenous in measurement and reporting of outcomes if they follow the CONSORT and EQUATOR Network guidelines [25]. Another limitation is that most studies did not describe their attrition rates. Of those that did, many had high loss, over the 20% considered acceptable for weight loss studies [26]. Attrition is an important consideration for generalizing the results of this and other studies, so more consistent reporting is necessary, along with improved strategies for retaining participants in weight loss studies.

The risk of bias across studies merited attention. There is a concern that authors and journal editors typically prefer to show weight/fat loss, and so there is a risk of positive publication bias in this field [26]. Indeed, while we know that when properly carried out, diet and PA studies do promote fat loss, many interventions suffer from attrition, loss of participant motivation, and weight/fat regain over time; most studies presented here do not include long-term follow-up data. It is likely that many studies with negative findings do not make it into the published literature. Unfortunately, there is no way to tell how many of those studies existed (particularly before the NIH clinical trials registry: http://clinicaltrials.gov/), but everyone in the weight loss research field should consider results with this caveat in mind [26]. Risk of bias in individual studies also merited attention. While, ideally, this would have been assessed, most studies did not provide enough information to make consistent or relevant judgments of bias (i.e., blinding is not possible in weight loss trials and attrition and funding sources were not always reported). Four out of the 28 studies (8.5%) reported funding conflicts of interest, indicating a low risk of bias. The risk of bias in the present review is also minimal, as the author had no sources of financial support in its creation.

In conclusion, % body fat in addition to or along with weight, BMI, and fat mass appears to be a useful, consistent, and meaningful measure of success in adults weight loss studies. It is recommended that researchers include it as a primary outcome measure in future studies.

## Figures and Tables

**Table 1:** Descriptive and technical components of the included studies.

Study (reference number)	Participants^*^	Baseline age (years) (mean ± SD or range)	Country and SES/race (if reported)	Degree of overweight for inclusion and comorbidities	Intervention length and setting	Intervention components	Body composition measure
[27]	19 women	43.2 ± 9.1	US: white, Hispanic, black	≥15 kg above ideal MetLife table weight, no comorbidities	16 week intervention, 1 year follow-up, university clinical research center	1200 cal/day, structured aerobic PA: 3x 45 min step aerobics classes/week	DEXA

[28]	111 women	35 ± 11.2 (19–50)	Cyprus	BMI >25, no comorbidities	18-week intervention plus 18-week follow-up, university clinical research center	1,500 ± 200 cal/day (50% carb, 30% fat, 20% protein) mod intensity PA 30–60 min/day; behavioral modification consult 1x/week	BIA

[29]	32 men and women	37.6 ± 4.4 (30–45)	Australia	BMI 27–32, no comorbidities	32 weeks, university clinical research center	Diet and PA general advice, cal counting resources, heart rate monitor, personalized goals	DEXA

[30]	44 women	54.7 ± 7.9 (postmenopausal)	US	BMI >30, postmenopausal, no comorbidities	24 weeks, university clinical research center	LEARN program: lifestyle nutrition, PA skills, weekly coaching, self-control training	BIA

[31] (aerobic treatment arm)	46 women	35.2 ± 7	US: white and black	BMI 27–30, no comorbidities	Hospital inpatient and outpatient; intervention until BMI <25: ~5.5 mo.	800 cal/day (meals provided), 40 min aerobic PA 3x/week	DEXA

[32]	105 men and women	44.9 ± 10.2 (18–65)	US: white and black	BMI 30–40, no comorbidities	2 years, multisite: university clinical research center/academic medical centers	1200–1500 cal/day (women), 1500–1800 cal/day (men), 20–50 min PA/walking 4x/week, behavioral counseling 1x/week	DEXA

[33] (aerobic treatment arm)	20 men and women	36 ± 7 (19–48)	US	Body weight >20% above “desired amount,” no comorbidities	8 weeks, university hospital GCRC	Liquid-formula diet: 1286 ± 281 cal/day, 30 min cycling (arms and legs) 3x/week, weekly nutrition counseling	Underwater weighing, BIA, and bi-/triceps skinfold

[34]	43 men and women	43 ± 12	Switzerland	BMI >30, no comorbidities	6 weeks, hospital inpatient	1000 cal/day low carb (15%) or moderate carb (45%), 1 h aerobic PA and 1 h underwater PA/day, nutrition education, “standard behavioral techniques”	BIA and triceps skinfold thickness

[35]	6 women	33 ± 8	US	>30% body fat, no comorbidities	16 weeks, university clinical research center	800 cal/day (low-fat diet), about 3 miles brisk walking/jogging 5 d/week, weekly diet education classes	Underwater weighing and triceps skinfold thickness

[36]	12 men and women	36 ± 6 (28–45)	US: mostly white	BMI 25–30, no comorbidities	24 weeks, university clinical research center	12.5% cal restriction (all food provided), 12.5% increase in energy expenditure structured PA (45–50 min cardio 5x/wk), weekly CBT group	DEXA

[37]	5 women	35 ± 4	US	37–50% body fat, no comorbidities	6 weeks, university hospital inpatient	800 cal/day (all food provided), about 4 miles daily walking	Underwater weighing

[38]	18 women	35 ± 7	US	130–160% “ideal body weight,” no comorbidities	12 weeks, university clinical research center	1200 cal/day constant or rotating 600–1800 cal/day, 5 d/week walking, behavior modification program	Underwater weighing

[39]	81 women	28 ± 1 (19–45)	Canada: multiracial (white, Indian, Asian primarily)	BMI 27–40, no comorbidities	16 weeks, university clinical research center	Individual weight maintenance cal level −500 cal/day (varying dairy levels), 5 d/week supervised aerobic PA (to burn 250 cal), 2 d/week strength training	DEXA

[40]	10 women	39.3 ± 5.4 (25–50)	The Netherlands	BMI >30, no comorbidities	8 weeks, no location noted	Diet: 3.5 mJ/day plus 1.4 mJ/day formula, 90 min each aerobics, fitness/strength 2 d/week	Underwater weighing

[41]	126 women	38.5 ± 8.5	US	BMI ≥30, no comorbidities	14 weeks, university clinical research center	1200 cal/day f/both: very low carb, high protein (63 : 7 : 30%), low carb, moderate protein (50 : 20 : 30%), high carb, low protein (55 : 15 : 30%), curves fitness program: 30 min circuit (strength and aero.) 3 d/week	DEXA

[42]	118 women	38.7 ± 7.5	US	BMI ≥30, no comorbidities	14 weeks, university clinical research center	1200 cal/day (phase 1) then 1600 cal (phase 2) diets varying macronutrients (carb/protein), curves fitness program: 30 min circuit (strength and aero.) 3 d/week	DEXA

[43]	34 men and women	18+	Ireland	BMI ≥28, any comorbidities except eye diseases and pregnancy	12 months, no location noted	1500 cal/day (women), 1800 cal/day (men) low-fat, 1 h/day PA classes, 1x/month motivational seminars	DEXA

[44] (diet + aerobic ex.)	9 women	Premenopausal (exact ages unknown)	US	>120% above MetLife table ideal weight or BMI ≥27, no comorbidities	12 weeks, university clinical research center	Matola food products provided, 30–50 min aerobic PA 3x/wk at 70–80% max HR, 1 h/wk group education	Underwater weighing

[44] (diet + aerobic + strength)	8 women	Same as above	Same as above	Same as above	Same as above	Diet and aerobic PA as above, plus 3x/week strength circuit	Same as above

[45]	90 women	18–55	US	BMI 27–40, no comorbidities	10 weeks, university clinical research center	Week 1: 1200 cal/day, weeks 2–10: 1600 cal/day, curves supervised PA program: 30 min circuit 3 days/week, met w/RD every 2 weeks	BIA

[46]	24 women	47.2 ± 1.3 (40–56)	US	BMI ≥26, no comorbidities	16 weeks, no location noted	Low (15%) or high (30%), pro: carb ratio, both: 1700 cal/day, ≥5 d/week walking, 2 d/week strength training, weekly nutritionist counsel.	DEXA

[47]	40 men and women	41 ± 7.7	US	Body fat ≥25% (men), 30% (wom), no comorbidities	12 weeks, university clinical research center	OPTIFAST: 420 cal/day, walking (60% HR) 3x/week to reduce 300 cal	BIA

[48]	22 men and women	43.0 ± 5.3 (29–50)	US	BMI 27–35, no comorbidities	24 weeks, independent outpatient clinical research center	500 cal deficit diet (meal replacements of 25–40% cals, plus supplemental foods), plus walking ≥5 d/week moderate intensity (300–500 cal), RD, PA couns. 1x/week	Air displacement plethysmography

[49]	100 women	20–65	Australia	BMI 27–40, no comorbidities	12 weeks, hospital outpatient GCRC	Food provided: 5600 kJ/day high protein (34%) or high carb (64%), ≥3x 30 min PA/week, nutritionist 1x/mo.	DEXA

[50]	10 women	38 ± 4.5 (21–47)	US	140%–180% weight f/height based on MetLife tables and body fat ≥35%, no comorbidities	12 weeks, university clinical research center	75% individuals' RMR: low-fat or low-carb diets, 45 min aerobic PA (60–60% max HR) 3x/week, nutrition and education meetings 1x/week	Doubly labeled water and appropriate calculations

[51]	48 women	53.8 ± 2.5 (postmenopausal)	Denmark	BMI ≥25, no comorbidities	12 weeks, university clinical research center	Up to 10 260 kJ portions of formula diet, small food supplementation, 1–1.5 h each aerobic and strength PA 3x/week	DEXA

[52]	20 men	25–50	The Netherlands	BMI ≥30, no comorbidities noted	12 weeks, outpatient site (no details noted)	Very low cal protein-enriched formula diet (Modifast): around 5 mJ/day), 1 hour low-intensity aerobic PA (40% HR) 4x/week, weekly meetings w/nutrition and exercise pros	underwater weighing

[53]	20 men and women	44.7 ± 13.0	Belgium	BMI 25–40, no comorbidities	24 week intervention + 24 week observation, outpatient hospital	600 cal deficit, 1 hour aerobic and strength training 2x/week, biweekly dietician	BIA

[54]	34 men and women	45.6 ± 9.0 (18–65)	US: mostly white, minority black	BMI 30–60, elevated lipids, no other comorbidities	24 weeks, outpatient clinical research center	Low-fat diet: 500–1000 cal/day deficit, ≥30 min aerobic PA 3 d/week, biweekly nutritionist meetings	BIA and DEXA (on a subset)

Note: university clinical research center: outpatient unless indicated otherwise.

Abbreviations. GCRC: General Clinical Research Center, BIA: bioelectrical impedance, DEXA: dual-energy X-ray absorptiometry, cal: calories, d/week: days per week, and h/day: hours per day.

^*^Only reporting participant data for the diet + aerobic PA intervention arm.

**Table 2:** Outcome data from the included studies: weight or BMI, body fat (BF) % or fat mass (FM), summary of weight or BMI and body fat measures, and attrition.

Study (reference number)	Baseline average weight (kg) ± SD and/or BMI (kg/m^2^) ± SD (range)	Baseline average % fat and/or fat mass (kg) ± SD (range)	Follow-up average weight (kg) ± SD and/or BMI (kg/m^2^) ± SD (range)	Follow-up average % fat and/or fat mass (kg) ± SD (range)	Average weight (kg) ± SD and/or BMI (kg/m^2^) change ± SD (range)	Average % fat or fat mass (kg) change ± SD (range or CI)	Summary/agreement of measures	Attrition (if reported)
[27]	Weight: 83.6 ± 8.6 BMI: 31.4 ± 3.7	% BF: 46.9 ± 3.6 FM: 39.3 ± 6.1	Not reported	% BF: 41.9 ± 4.3	Weight loss: −8.3 ± 3.1	% BF: −5.0 ± 3.9 FM: −7.4 ± 3.7	Weight and fat measures decreased significantly. Weight loss mostly accounted for by FM.	

[28]	Weight: 79.8 ± 11.8 BMI: 29.1 ± 4.8	% BF: 39.3 ± 7.5	Weight: 62.5 ± 8.3 BMI: ±24 ± 3.5	% BF: 29.3 ± 7.0	Weight loss: −17.3 (CI: 15.7–18.9) (=−22%) BMI: −5.1 (CI: −3.8–−6.4) (=−17.5%)	% BF: −10.0 (CI: 9–11)	All weight or BMI and fat measures decreased significantly.	

[29]	Weight: 87.2 ± 12.6 BMI: 29.3 ± 1.6	% BF: 35.5 ± 6.0	Not reported	Not reported	Weight loss: −6.1 ± 0.6 (=−7.1%)	FM: −5.9 ± 0.6	Weight and fat measures decreased significantly. Weight loss mostly accounted for by FM.	20%

[30]	Weight: 96.4 ± 16.0 BMI: 36.4 ± 5.5	% BF: 46.2 ± 5.6 FM: 45.8 ± 12.3	Weight: 88.6 ± 15.7 BMI: 33.6 ± 5.3	% BF: 44.5 ± 6.1 FM: 39.6 ± 11.8	Weight loss: −7.8 ± 15.9 BMI: −2.8 ± 5.4	% BF: −2.4 ± 5.8 FM: −3.6 ± 12.0	Weight or BMI and fat measures decreased significantly.	25%

[31]	Weight: 76.9 ± 6.6 BMI: 28.5 ± 1.5	% BF: ±44.1 ± 3.8 FM: 34.1 ± 5.0	Weight: 64.3 ± 6.1 BMI: 23.8 ± 1.1	% BF: 33.9 ± 4.5 FM: 22.0 ± 4.6	Weight loss: −12.5 ± 2.2 BMI: −4.7 ± 1.3	% BF: −10.2 ± 4.1 FM: −12.1 ± 4.8	Weight or BMI and fat measures decreased significantly. Weight loss mostly accounted for by FM.	

[32]	Weight: 103.5 ± 14.4 BMI: 36.1 ± 3.5	FM: 40.4 ± 7.8	Not reported	Not reported	Weight loss: −7.4 (CI: −9.1–−5.6)	FM: −3.8 (−5.0–−2.6)	Weight and fat measures decreased (not noted whether differences were significant).	32%

[33] (aerobic treatment arm)	Weight: 96.0 ± 23.0	FM: 38.4 ± 12.5	Weight: 86.4 ± 19.8	FM: 31.2 ± 11.0	Weight loss: −9.6 ± 4.5	FM: −7.2 ± 3.0	Weight or BMI and fat measures decreased significantly. Weight loss mostly accounted for by FM.	

[34]	Weight: 104.5 ± 5.5 BMI: 39.5 ± 7.0	FM: 44.0 ± 2.5	Weight: 97.0 ± 3.5	FM: 36.0 ± 2.0	Weight loss: −7.5 ± 4.5	FM: −8.0 ± 2.2 (=−17.2%)	Weight or BMI and fat measures decreased significantly. Weight loss mostly accounted for by FM.	

[35]	Weight: 87.7 ± 22.6 BMI: 32.2 ± 7.8	% BF: 38.4 ± 6.0	Weight: 74.8 ± 21.4	% BF: 30.3 ± 7.1	Weight loss: −8.2 ± 0.7	% BF: −8.1 ± 6.5	Weight and fat measures decreased significantly. Weight loss mostly accounted for by FM (91% loss was fat).	28%

[36]	Weight: 81.9 ± 10.5 BMI: 27.5 ± 1.6	% BF: 32.6 ± 7.6	Not reported	Not reported	Weight loss: −8.0 ± 2.0 (=−10.0%)	% BF: −25% change in % BF ± 3	Weight and fat measures decreased significantly.	4%

[37]	Weight: 100.2	% BF: 45.0%	Weight: 92.6	% BF: 41.8	Weight loss: −7.6	% BF: −3.2	Weight and fat measures decreased significantly. Diet and PA group lost significantly more fat and significantly less fat-free mass than non-PA subjects.	

[38]	BMI: 30.5 ± 3	% BF: 43.9 ± 1.1	Not reported	% BF: 38.5 ± 1.3	Weight loss: −8.6 ± 0.9	% BF: −4.5% FM: −7.0 ± 0.6	Diet + PA group lost significantly more weight than non-PA group; diet + PA group had significantly greater decrease in % BF than non-PA group. PA: 86% of weight loss from fat versus 73% f/non-PA group	20%

[39]	Weight: 85.2 kg ± 2.1 BMI: 31.6 ± 0.6	% BF: 40.2 ± 0.7 FM: 34.5 ± 1.3	Not reported	Not reported	Weight loss: −4.3 ± 0.7 BMI decrease: −1.8 ± 0.3	% BF: −1.3 ± 0.2 FM: −1.7 ± 0.5	Weight or BMI and fat measures decreased significantly. Fat loss more significant than weight loss.	10% at halfway point of study

[40]	Weight: 90.4 ± 2.9 BMI: 32.4 ± 1.3	% BF: 41.6 ± 1.5 FM: 38.0 ± 2.9	Not reported	Not reported	Weight loss: −9.1 ± 1.1	FM: −7.8 ± 0.82	Weight and fat measures decreased significantly. Weight loss mostly accounted for by FM: percentage of weight lost as fat significantly different for diet + PA group (88.6 ± 5.1%), versus diet only group (77.1 ± 4.0%).	

[41]	Weight: 95.7 ± 16.6 BMI: 35.3 ± 5.7	FM: 39.3 ± 10	Not reported	Not reported	Weight loss: −5.4 (−7.0–3.0)	% BF: −1.9% (−2.6–−1.3) FM: −3.3 (−4.0–−2.0)	Weight and fat measures decreased significantly.	33%

[42]	Weight: 94.3 kg ± 16.0 BMI: 35.7 ± 6.0	% BF: 45.1 ± 4.2 FM: 40.0 ± 9.7	Weight: 90.1 ± 15.6	% BF: 43.2 ± 4.7 FM: 36.6 ± 9.2	Weight loss: −4.2 ± 15.8	% BF: −1.9 ± 4.5 FM: −3.4 ± 9.5	Weight and fat measures decreased significantly. Weight loss mostly accounted for by FM.	35%

[43]	Weight: 96.5 ± 20.2 BMI: 35.3 ± 6.5	% BF: 41.9 ± 9.4 FM: 40.3 ± 12.8	Weight: 93.3 ± 21.2 BMI: 34.2 ± 6.6	% BF: 41.2 ± 8.9 FM: 38.6 ± 12.6	Weight loss: −2.9 ± 20.7 BMI: −1.1 ± 6.5	% BF: −0.7 ± 9.2 FM: −0.9 ± 12.7 (=−2.3%)	Weight decreased significantly; body FM was “borderline significant” (*P* = 0.053). BMI and % BF declined more in intervention group than control, no significant differences between groups.	

[44] (diet + aerobic ex.)	Not reported	Not reported	Not reported	Not reported	Weight loss: −6.8	% BF: −8.0	Weight and fat measures decreased significantly.	

[44] (diet + aerobic + strength)	Not reported	Not reported	Not reported	Not reported	Weight loss: −7.0	% BF: −4.3	Results same as above. Weight loss through diet was not altered by aerobic or aerobic + strength training, but diet + PA increased muscle during weight loss.	

[45]	Weight: 89.2 ± 12.0 BMI: 33.1 ± 4.0	% BF: 44.1 ± 4.0 FM: 36.8 ± 8.0	Weight: 86.1 ± 11.0 BMI: 32.0 ± 4.0	% BF: 43.1 ± 5.0 FM: 34.5 ± 7.0	Weight loss: −3.1 ± 11.5 BMI: −1.1 ± 4.0	% BF: −1.0 ± 4.5 FM: −2.3 ± 7.5	Weight or BMI and fat measures decreased significantly. Weight loss mostly accounted for by FM.	

[46]	Weight: 82.9 ± 3.6 BMI: 30.8 ± 1.5	FM: 38.6 ± 2.9	Weight: 74.7 ± 3.3	FM: 31.4 ± 2.6	Weight loss: −8.2 ± 3.4	FM: −7.2 ± 0.7	Weight or BMI and fat measures decreased significantly. Weight loss mostly accounted for by FM. More fat loss associated with high protein diet + PA	

[47]	Weight: 106.0 ± 25.6	% BF: 44.4 ± 5.6 FM: 47.6 ± 15.2	Weight: 90.7 ± 21.6	% BF: 35.3 ± 7.3 FM: 32.7 ± 12.7	Weight loss: −15.3 ± 1.1	% BF: −9.1 ± 0.5 FM: −14.9 ± 0.8	Weight and fat measures decreased significantly. Weight loss mostly accounted for by FM.	

[48]	Weight: 88.8 ± 3.3 BMI: 31.8 ± 0.7	% BF: 45.1 ± 1.3 FM: 40.2 ± 2.1	Weight: 81.7 ± 2.9 BMI: 28.0 ± 1.5	% BF: 39.7 ± 1.6 FM: 32.6 ± 1.9	Weight loss: −7.1 ± 3.1 (=−8%) BMI: −3.8 ± 1.1 (=−12%)	% BF: −5.4 ± 1.4 FM: −7.6 ± 2.0	Weight and fat measures decreased significantly. Weight loss mostly accounted for by FM. Diet + PA group lost more fat than PA only group.	48.8% in diet + PA group

[49]	Weight: 86.5 ± 12.0 BMI: 32.5 ± 5.0	FM: 42.0 ± 1.1	Not reported	FM: 36.8 ± 1.1	Weight loss: −7.6 ± 0.4	FM: −5.1 ± 0.5	Weight and fat measures decreased significantly. Weight loss mostly accounted for by FM.	16%

[50]	Weight: 92.1 ± 8.8	% BF: 44.0 ± 3.2	Not reported	Not reported	Weight loss: −10.5 ± 3.3	FM: −8.8 ± 2.1	Weight and fat measures decreased significantly. Weight loss mostly accounted for by FM.	25%

[51]	Weight: 78.1 ± 10.3	FM: 31.9 ± 6.2	Not reported	Not reported	Weight loss: −10.3 ± 3.0	FM: −9.6 ± 2.7	Weight and fat measures decreased significantly. Weight loss mostly accounted for by FM. Diet + PA group lost no lean tissue mass.	2.5%

[52]	Weight: 101.9 ± 11.2 BMI: 32.6 ± 2.5	% BF: 33.5 ± 4.2 FM: 34.2 ± 6.1	Weight: 86.7 ± 9.3 BMI: 27.8 ± 2.5	% BF: 25.0 ± 5.0 FM: 21.7 ± 5.2	Weight loss: −15.2 ± 6.3 BMI: −4.8 ± 2.5	% BF: −8.5 ± 4.7 FM: −12.5 ± 5.6	Weight or BMI and fat measures decreased significantly. Weight loss mostly accounted for by FM.	

[53]	Weight: 94.5 ± 11.7 BMI: 33.1 ± 3.4	Not reported	Not reported	Not reported	Weight loss: −6.6 ± 6.4 BMI: −2.3 ± 2.1	% BF: −4.0 ± 4.1	Weight or BMI and fat measures decreased significantly.	23%

[54]	Weight: 95.7 ± 18.0 BMI: 33.9 ± 5.3	Not reported	Not reported	Not reported	Weight loss: −6.5 (CI: −4.6–−8.4)	% BF: −2.8 (CI: −1.9–−3.9) FM: −4.8 (CI:−3.2–−6.3)	Weight or BMI and fat measures decreased significantly. Weight loss mostly accounted for by FM.	24–43% (different groups)

Note: outcome data are presented for the longest follow-up time available. CI: 95% confidence interval. When body weight or BMI and/or body composition outcome measures were not reported, the data were not imputed as long as the change measures were intact and reported; in those cases “not reported” is presented. Significance indicates differences compared to baseline values and to diet only/control group(s) when indicated.
